# Development of a novel micro-bead force spectroscopy approach to measure the ability of a thermo-active polymer to remove bacteria from a corneal model

**DOI:** 10.1038/s41598-021-93172-1

**Published:** 2021-07-01

**Authors:** J. Pattem, T. Swift, S. Rimmer, T. Holmes, S. MacNeil, J. Shepherd

**Affiliations:** 1 School of Clinical Dentistry, University of Sheffield, Sheffield, UK; 2 Polymer and Biomaterials Chemistry Laboratories, School of Chemistry and Biosciences, University of Bradford, Bradford, UK; 3 Department of Oncology and Metabolism, School of Medicine, University of Sheffield, Sheffield, UK; 4 Department of Materials Science and Engineering, Faculty of Engineering, University of Sheffield, Sheffield, UK; 5 National Centre for Molecular Hydrodynamics, and, Soft Matter Biomaterials and Bio-Interfaces, University of Nottingham, The Limes Building, Sutton Bonington Campus, Sutton Bonington, Leicestershire, LE12 5RD UK

**Keywords:** Nanoscale biophysics, Biophysics, Microbiology, Diseases, Health care, Medical research, Chemistry, Materials science, Nanoscience and technology

## Abstract

Microbial keratitis occurs from the infection of the cornea by fungi and or bacteria. It remains one of the most common global causes of irreversible blindness accounting for 3.5% (36 million) of blind people as of 2015. This paper looks at the use of a bacteria binding polymer designed to bind *Staphylococcus aureus* and remove it from the corneal surface. Mechanical unbinding measurements were used to probe the interactions of a thermo-active bacteria-binding polymer, highly-branched poly(N-isopropyl acrylamide), functionalised with modified vancomycin end groups (HB-PNIPAM-Van) to bacteria placed on rabbit corneal surfaces studied *ex-vivo*. This was conducted during sequential temperature phase transitions of HB-PNIPAM-Van-*S. aureus* below, above and below the lower critical solution temperature (LCST) in 3 stages, in-vitro, using a novel micro-bead force spectroscopy (MBFS) approach via atomic force microscopy (AFM). The effect of temperature on the functionality of HB-PNIPAM-Van-*S. aureus* showed that the polymer-bacteria complex reduced the work done in removing bacterial aggregates at T > LCST (*p* < 0.05), exhibiting reversibility at T < LCST (*p* < 0.05). At T < LCST, the breaking force, number of unbinding events, percentage fitted segments in the short and long range, and the percentage of unbinding events occurring in the long range (> 2.5 µm) increased (*p* < 0.05). Furthermore, the LCST phase transition temperature showed 100 × more unbinding events in the long-range z-length (> 2.5 µm) compared to *S. aureus* aggregates only. Here, we present the first study using AFM to assess the reversible mechanical impact of a thermo-active polymer-binding bacteria on a natural corneal surface.

## Introduction

Microbial keratitis is a condition arising from the infection of the cornea by microbes, most commonly fungi and bacteria^1–3^ and remains one of the most common global causes of irreversible blindness^4,5^. Manifesting as corneal ulceration and opacification in its latter stages^6,7^ microbial keratitis is up to 80 times more common in India compared to Western countries^8,9^ and particularly affects poorer and rural farming communities^10^. These communities are also more likely to exhibit corneal damage and infection from eye rubbing, due to the frequent handling of dirty water and exposure to dust, insecticides, and fertilizers due to lack of protective equipment^10,11^. Resultant scarring from abrasion and prolonged microbial infection requires difficult and costly surgery to resolve^12,13^. India’s rural communities are also less likely to detect this disease in Its preliminary stages, due to insufficient access to clinicians for effective diagnosis^14,15^. Therefore, treatment with broad-spectrum antibiotics is often applied^16,17^, contributing to increased antimicrobial resistance (AMR) in the microbial community, reducing the effect current antibiotics have on this potentially debilitating disease and others^18,19^. Currently, *Staphylococcus aureus* is the most common Gram-positive bacteria involved in microbial keratitis^7,20^ and, resistance rates of more than 30% to fluoroquinolone and methicillin antibiotics have been reported^21^.

Researchers are increasingly seeking approaches to prevent corneal infections that do not promote AMR and improve antimicrobial stewardship^22,23^. These can either be advances in early stage detection for targeted therapeutics^24,25^, or progress in the removal or killing of the infectious organisms at the area without traditional use of antibiotics^26,27^. Removal poses a significant challenge to clinicians, as complex interventions are required to reduce pathogenic dispersion to the cornea^22,28^. This becomes an even greater challenge as non-AMR based approaches must not further damage the corneal surface in using high concentrations of surfactant-based^26,29,30^, polymer^31,32^, peptide or lipopeptide amphiphile interventions^33,34^.

**Figure 1:**
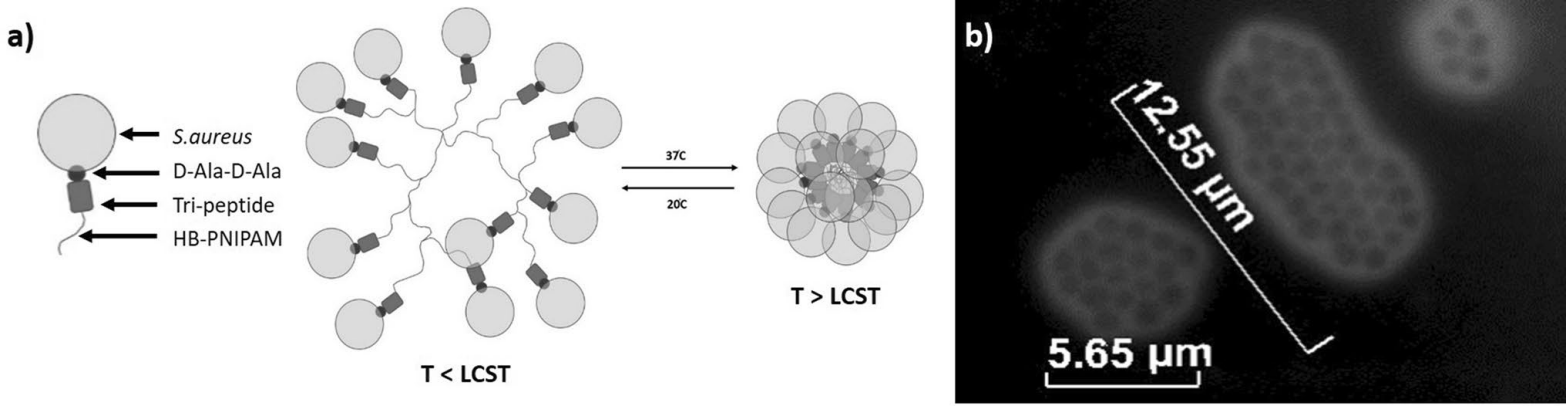
Showing (**a**) Schematic of *S. aureus*-Van-HB-PNIPAM above (left) and below (right) the LCST exhibiting contraction during its desolvation phase shift and (**b**) Fluorescence microscope image showing *S. aureus*-Van-HB-PNIPAM at T > LCST stained with Syto-9.

One such novel intervention is the use of thermo-sensitive, hyper-branched poly(N-isopropylacrylamide), functionalised with modified antibiotics at its terminals, e.g. Vancomycin (HB-PNIPAM-Van)^22,35–38^ or polymixin (HB-PNIPAM-PMX)^39^. The antibiotic terminals can attach to dispersed bacteria via peptide ligand receptors^22,35,36,38^ shown in Fig. 1a. HB-PNIPAM is known for its ability for segments of the polymer chain to transition from a swollen phase (solvated), to a collapsed phase (de-solvated) across the lowest critical solution temperature (LCST)^40^. Binding bacteria affects the energetics of this transition, so as a result, -HB-PNIPAM-X’s solvation, a resultant chain volume, are responsive to both bacterial loading and the environmental temperature^22,35–38^, as shown in Fig. 1a. This phase transition LCST can also be tailored to occur at physiologically relevant temperatures, e.g. 37.5 °C^40^. HB-PNIPAM-Van has shown promise in aggregating dispersed *S. aureus* bacteria shown in Fig. 1b, creating large, localised aggregates on corneal abrasions, in-vitro, via fluorescence imaging^22^. However, to date there are no reports on the effect this has on the mechanical unbinding properties of bacterial aggregates to a corneal surface. One technique that can be useful in assessing this affect is atomic force microscopy (AFM)^41^, particularly, the use of Single-cell^42,43^ (SCFS) and Micro-bead force spectroscopy (MBFS)^44^. This technique enables single bacteria or whole biofilms respectively to be pulled away from a surface of interest via a modified and functionalised cantilever^43,44^. Unbinding properties from resulting force-distance curves such as energy dissipation (work done) and unbinding events can be assessed^42^. It has enabled the adhesion characteristics of *S. aureus* on implant surfaces^43^ to be elucidated and a number of other bacteria to clinical surfaces of interest^45^. Furthermore, biofilm-surface interactions investigated using this technique have identified differences in early and proliferated biofilm adhesion characteristics under in-vitro conditions with success^44^.

This investigation aims to use micro-bead force spectroscopy (MBFS) to determine the effect HB-PNIPAM-Van has on the unbinding characteristics of *S. aureus* aggregates from a corneal surface, in-vitro. Particularly, assessing the effect of sequential phase transitions at physiologically relevant LCST, previously un-reported.

## Materials and methods

### Bacterial cultures

For all experiments, a clinical isolate strain *of S. aureus* (S235) was used as a representative Gram-positive corneal infection pathogen. Bacterial cultures were maintained on brain heart infusion (BHI) agar, and before use, bacteria were incubated in BHI broth (Sigma-Aldrich) at 37 °C overnight, to be used the next day. 1 ml of bacteria in suspension in BHI broth were then pipetted into an Eppendorf and centrifuged at 3500 g for 3 min to obtain a pellet. The BHI was removed and washed with PBS (Lonza, BioWhittaker, UK). Washing and re-suspension was performed 3 times and the concentration of bacteria was adjusted via manual counting using a cell counting chamber (Neubauer, EMS, UK) to provide 1 ml of *S. aureus* in PBS at the desired concentration of 6 CFU/ml of bacteria. Syto-9 (Sigma-Aldrich) was used to stain *S. aureus* and HB-PNIPAM-Van-*S. aureus* for fluorescence microscopy only.

### HB-PNIPAM-Van *S. aureus* solutions

HB-PNIPAM-Van solutions were prepared using the protocol developed by Doroshenko and co-workers^22^. 0.05 g of HB-PNIPAM-Van^46^ was dissolved in 10 ml of PBS, resulting in a 5 mg/mL stock solution. 50 µL of bacteria suspension and 50 µL of HB-PNIPAM-Van solution was added to a 96-well plate in triplicate. Also, 50 µL of HB-PNIPAM-Van with 50 µL PBS and 50 µL of bacteria suspension with PBS were used as controls. These were then incubated overnight at 37 °C.

### Atomic force microscopy analysis

#### Probe modification and functionalisation

**Figure 2:**
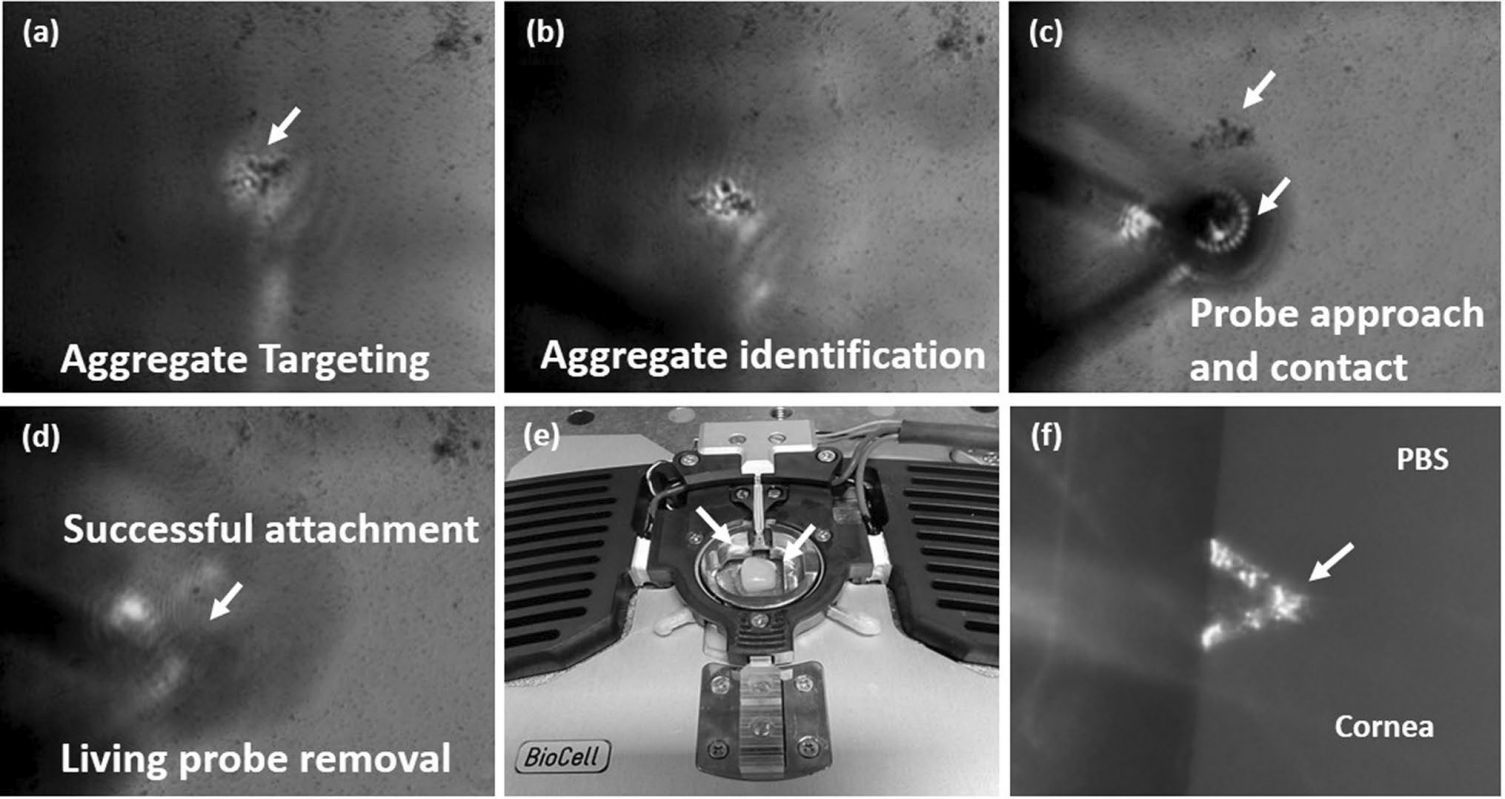
Showing (**a**) AFM Topview light microscope image of *S. aureus*-Van-HB-PNIPAM target adsorbed to glass slide under PBS conditions, (**b**) 50 µm modified spherical AFM probe functionalised with poly-DOPA approaching target (**c**) 50 µm modified spherical AFM probe functionalised with poly-DOPA contacting target, (**d**) Removal of 50 µm functionalised poly-DOPA-*S. aureus*-Van-HB-PNIPAM spherical AFM probe, (**e**) Rabbit cornea in AFM Temperature cell and (**f**) 50 µm functionalised *S. aureus*-Van-HB-PNIPAM spherical AFM probe approaching rabbit cornea in PBS at 22 ֯C.

A JPK Nanowizard 3 AFM (JPK Instruments, Ltd) was used to modify NPO-10 tip-less cantilevers (Bruker Ltd, France). Cantilevers were modified with 50 µm borosilicate spheres (Whitehouse Scientific, UK) using a UV curing resin (Loctite, UK). Modified cantilevers were cured under UV light for 5 min. Cantilevers were then functionalised by exposure to 4 mg/mL poly-dopamine (poly DOPA) (Sigma-Aldrich, UK) in Tris Buffer (pH 8) (Sigma-Aldrich, UK) for 1 h and subsequently dried with N_2_ gas (n = 9). Successfully modified cantilevers were calibrated before analysis generating a spring constant of 0.03 ± 0.05 N/m. To create the living probe, HB-PNIPAM-Van-*S. aureus* was physiosorbed to a glass slide for one hour and rehydrated with PBS. Aggregates attached to the glass surface shown in Fig. 2a,b were collected onto the probe by approaching and indenting them with 1 nN force, holding for 30 s as shown in Fig. 2c. The probe, now termed the “living probe” was then removed to determine successful attachment and was now ready to use, shown in Fig. 2d. This was repeated on bacterial suspensions without HB-PNIPAM-Van serving as a control.

#### In-vitro eye model

**Figure 3:**
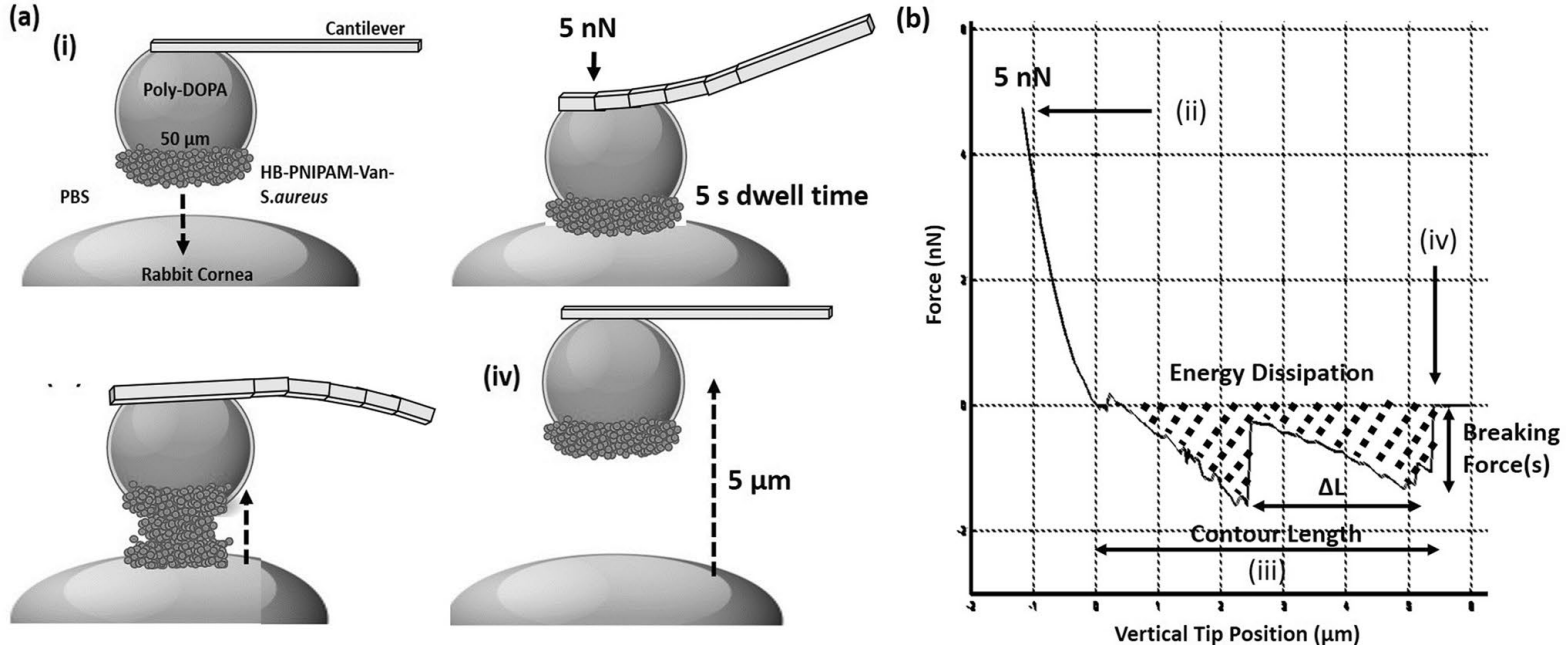
Showing a schematic of (**a**), (i) 50 µm functionalised poly-DOPA-*S. aureus*-Van-HB-PNIPAM spherical AFM probe approaching cornea, (ii), contact with rabbit cornea, (iii) adhesive removal from cornea, (iv) complete removal from cornea and (**b**) showing corresponding AFM retract force-distance curve for (ii), (iii) and (iv).

Fresh rabbit corneas (n = 9) were harvested from female New Zealand White rabbits (Envigo) sacrificed for an unrelated study and placed in PBS (Lonza, BioWhittaker, UK) solution ready for experimentation. All animal tissues used in this study were obtained from animals licenced and strictly regulated by the UK Home Office under the Animals (Scientific Procedures) Act 1986 and all licenced work was approved by the University of Sheffield Animal Welfare and Ethical Review Body. All experiments were performed in accordance with and complied with the ARRIVE guidelines. Fresh corneas were used immediately without storage. These were then cut using a scalpel to produce 1 cm x 1 cm × 0.5 x cm sample with the cornea apex in the centre, enabling the probe and specimen to be fully submerges in PBS within the Bio-cell. Cornea specimens were then secured to a circular glass coverslip using an araldite composite resin and placed into a temperature cell (Bio-cell, JPK Instruments, Bruker, France) shown in Fig. 2e. The living probe was then placed directly above the cornea apex and the cell was completely submerged in PBS at stage 1 (22 °C, T_1_ < LCST) shown in Fig. 2f. Indentations (n = 100) were performed at 5 nN force with a 5 s dwell time, in a 10 µm × 10 µm area in a 4 × 4 array on the cornea. After which, the living probe was retracted from the surface while remaining in the PBS solution and heated in stage 2 (40 °C, T_2_ < LCST) for 30 min. The same indentation protocol (n = 100) was repeated in an adjacent 10 µm × 10 µm area in the same manner. After which, the temperature cell was cooled back down at stage 3 (22 °C, T_3_ < LCST) for 30 min and the same indentation protocol was repeated on another adjacent area. Figure 3a(i–iv) shows a schematic of the living probe indenting a cornea with a resulting force-distance retract curve shown in Fig. 3b. The experimental design was split into 3 stages using the same living probe during the indentation protocol in triplicate. Three fresh corneas were tested per day using the same bacterial culture age, creating 1 HBPNIPAM-Van-*S. aureus* probe, 1 *S. aureus* probe and 1 poly-DOPA only probe. This was repeated 3 times to generate n = 3 per probe group ensuring the same bacterial culture age was used cross all probes, on fresh corneas.

#### Data and analysis

Energy Dissipation (× 10^6^ J), the work done in probe removal, was collected from the area of the resulting retract force curves using dedicated software (JPK Data Processing Software, JPK Instruments, Bruker, France). Statistical analysis was performed using a series of Moods Median tests and 1-way ANOVAs comparing each experimental group temperature stages and controls (*S. aureus* only). Breaking force (nN), was calculated from the average of large non-specific and specific adhesion force events across the retract z-length. The number of unbinding events (fitted segments), their percentage in the long range (FSLR) (> 2.5 µm %) and short range (FSSR) (< 2.5 µm %), the distance between unbinding events, ΔL (nm) and contour length (total length of aggregate) (µm) were analysed using the worm-like-chain (WLC) model. Including, the percentage of unbinding events occurring beyond half of the indentation z-length (2.5 µm), X_max_ < 2.5 µm (%).

## Results

### Quantitative mechanics

#### Energy dissipation

Table 1 shows median and interquartile ranges of energy dissipation (× 10^6^ J) for HB-PNIPAN-Van-*S. aureus, S. aureus* only and poly-DOPA only functionalised AFM probes, during removal from the corneal surface. At stage 1 (T_1_ < LCST, 22 °C), HB-PNIPAM-Van-*S. aureus* aggregates exhibited significantly greater energy dissipation to remove from the living probe from the corneal surface (212 ± 174 × 10^6^ J) compared to *S. aureus* aggregates only (155 ± 89 × 10^6^ J) (*p* < 0.05). When PBS in the in-vitro environment was heated in stage 2 (T_2_ > LCST, 40 °C), *S. aureus* only aggregates exhibited a slight but not significant increase in energy dissipation shown in Table 1. Conversely, HB-PNIPAM-Van-*S. aureus* aggregates exhibited a significant decrease in energy dissipation, changing from 212 ± 174 × 10^6^ J to 174 ± 154 × 10^6^ J (*p* < 0.05), shown in Table 1. At stage 3 (22 °C, T_3_ < LCST), both HB-PNIPAM-Van-*S. aureus* aggregates and *S. aureus* aggregates exhibited a significant increase in energy dissipation (*p* < 0.05) shown in Table 1, which were not significantly different from each other (*p* > 0.05).

**Table 1:** Showing the unbinding mechanical properties of HB-PNIPAM-Van-*S. aureus*, *S. aureus* and poly-DOPA functionalised probes during removal from the corneal surface using AFM.

Specimen/Stage	Energy dissipation (× 10^6^ J)	Breaking force (nN)	No. of fitted segments (FS)	ΔL (nm)	Contour length (μm)	FS_SR_ (%)	FS_LR_ (%)	X_max_ > 2.5 μm (%)
HB-PNIPAM-Van-*S. aureus* Stage 1	212 ± 174	0.5 ± 0.4	1206	109 ± 105	1.9 ± 1.3	69	31	10.78
HB-PNIPAM-Van-*S. aureus* Stage 2	174 ± 154*	0.8 ± 0.6*	2601	75 ± 68*	2.1 ± 1.2*	64	36	9.02
HB-PNIPAM-Van-*S. aureus* Stage 3	232 ± 151*	0.7 ± 0.5*	2731	73 ± 69	1.1 ± 0.8*	92	8	7.72
*S. aureus* Stage 1	155 ± 89	0.7 ± 0.6	3194	35 ± 30	1.6 ± 1.1	82	18	1.37
*S. aureus* Stage 2	167 ± 167	0.8 ± 0.7	3482	36 ± 31	1.6 ± 1.1	80	20	0.06
*S. aureus* Stage 3	184 ± 150*	0.9 ± 0.7	4776	26 ± 18	1.3 ± 0.9	89	11	0
Poly-DOPA Stage 1	5 ± 1	–	–	–	–	–	–	–
Poly-DOPA Stage 2	5 ± 2	–	–	–	–	–	–	–
Poly-DOPA Stage 3	5 ± 2	–	–	–	–	–	–	–

*p < 0.05.

#### Breaking force

Corresponding breaking force (nN) was obtained from the average specific and non-specific adhesion events from each individual force-curve, at each temperature stage for both HB-PNIPAM-Van-*S. aureus* and *S. aureus* aggregates only. Again, HB-PNIPAM-Van-*S. aureus* exhibited functionality with a significant increase and subsequent decrease in breaking force during the temperature phase transitions from Stage 1 to 2 and to 3, characterised by the values of 0.5 ± 0.4 nN and 0.8 ± 0.6 nN, 0.7 ± 0.5 nN respectively (*p* < 0.05), shown in Table 1. *S. aureus* aggregates exhibited no significant change in breaking force across all temperature stages (*p* > 0.05).

### Quantitative unbinding using worm-like-chain model

#### Distance between unbinding events (ΔL)

The distance between unbinding events (ΔL) during probe removal from the cornea were 2–3 × greater for HB-PNIPAM-Van-*S. aureus* compared to *S. aureus* probes only at all stages (*p* < 0.05), shown in Table 1. During the phase transition LCST from stage 1 to 2, HB-PNIPAM-Van-*S. aureus* exhibited a significant reduction ΔL from 109 ± 105 nm to 75 ± 68 nm (*p* < 0.05). No significant change in ΔL was found after transition back to T_3_ < LCST for HB-PNIPAM-Van-*S. aureus*. There was no significant change in ΔL for *S. aureus* aggregates at all temperature stages.

#### Contour length

HB-PNIPAM-Van-*S. aureus* aggregates showed a significant increase (*p* < 0.05), decrease ((*p* < 0.05) and subsequent increase (*p* < 0.05) in contour length (total aggregate length) during unbinding from the corneal surfaces at stager 1, 2 and 3 respectively shown in Table 1. *S. aureus* aggregates did not significantly change (*p* > 0.05) over the entire temperature regime. Also, contour lengths for HB-PNIPAM-Van-*S. aureus* were significantly larger than *S. aureus* only (*p* < 0.05).

#### Number of fitted segments (FS), percentage of FS in the short range (FSSR) and long range (FSLR)

**Figure 4:**
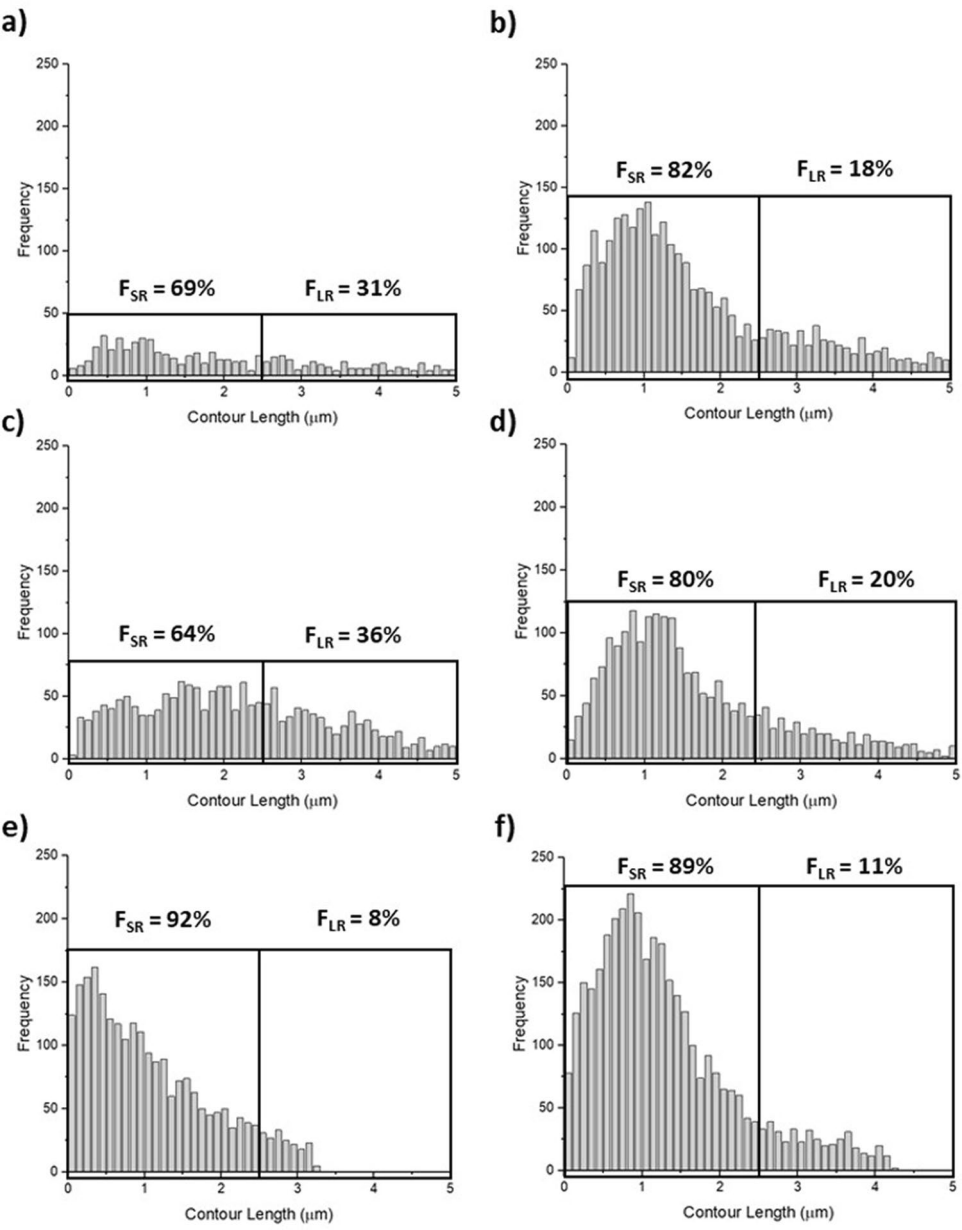
Showing histograms and percentages of long range (> 2.5 µm) (FSLR) and short range (< 2.5 µm) (FSSR) fitted segments (total number of unbinding events) for (**a**) HB-PNIPAM-Van-*S. aureus* at stage 1, (**b**) *S. aureus* at stage 1, (**c**) HB-PNIPAM-Van-*S. aureus* at stage 2, (**d**) *S. aureus* at stage 2, (**e**) HB-PNIPAM-Van-*S. aureus* at stage 3 and (**f**) *S. aureus* at stage 3.

The number of fitted segments i.e. the total number of unbinding events in the retract curve was significantly less across all temperature stages for HB-PNIPAM-Van-*S. aureus* compared to *S. aureus* only (*p* < 0.05) shown in Table 1, with histograms shown in Fig. 4. HB-PNIPAM-Van-*S. aureus* exhibited a 5% decrease in FSSR from stage 1 to stage 2 above the LCST, with a subsequent 28% increase at stage 3, below the LCST shown in Table 1. *S. aureus* aggregates exhibited a higher percentage of FSSR at stage 1 and 2 compared to HB-PNIPAM-Van-*S. aureus* aggregates, with a 2% decrease with increasing temperature above the LCST. At stage 3, a 9% increase in FSSR is shown in Table 1 from stage 2 to 3 for *S. aureus* aggregates.

The percentage of fitted segments in the long range (% FSLR) were significantly greater for HB-PNIPAM-Van-*S. aureus* aggregates compared to *S. aureus* aggregates only, at stage 1 and 2 (*p* < 0.05), shown in Fig. 4a,c. HB-PNIPAM-Van-*S. aureus* exhibited a 5% increase in FSLR at its phase transition temperature (stage 1 to 2) shown in Fig. 4a,c, consequently reducing by 28% at stage 3 shown in Fig. 4e and Table 1. A similar pattern is obtained for *S. aureus* aggregates only, albeit much less severe, exhibiting an FSLR percentages increase of 2%, shown in Fig. 4b,d and Table 1, from stage 1 to 2. After this, a reduction of 9% from stage 2 to 3 is shown in Table 1 and Fig. 4d,f for *S. aureus* aggregates only.

#### Percentage unbinding events occurring beyond 2.5 µm (X_max_ > 2.5 µm)

**Figure 5:**
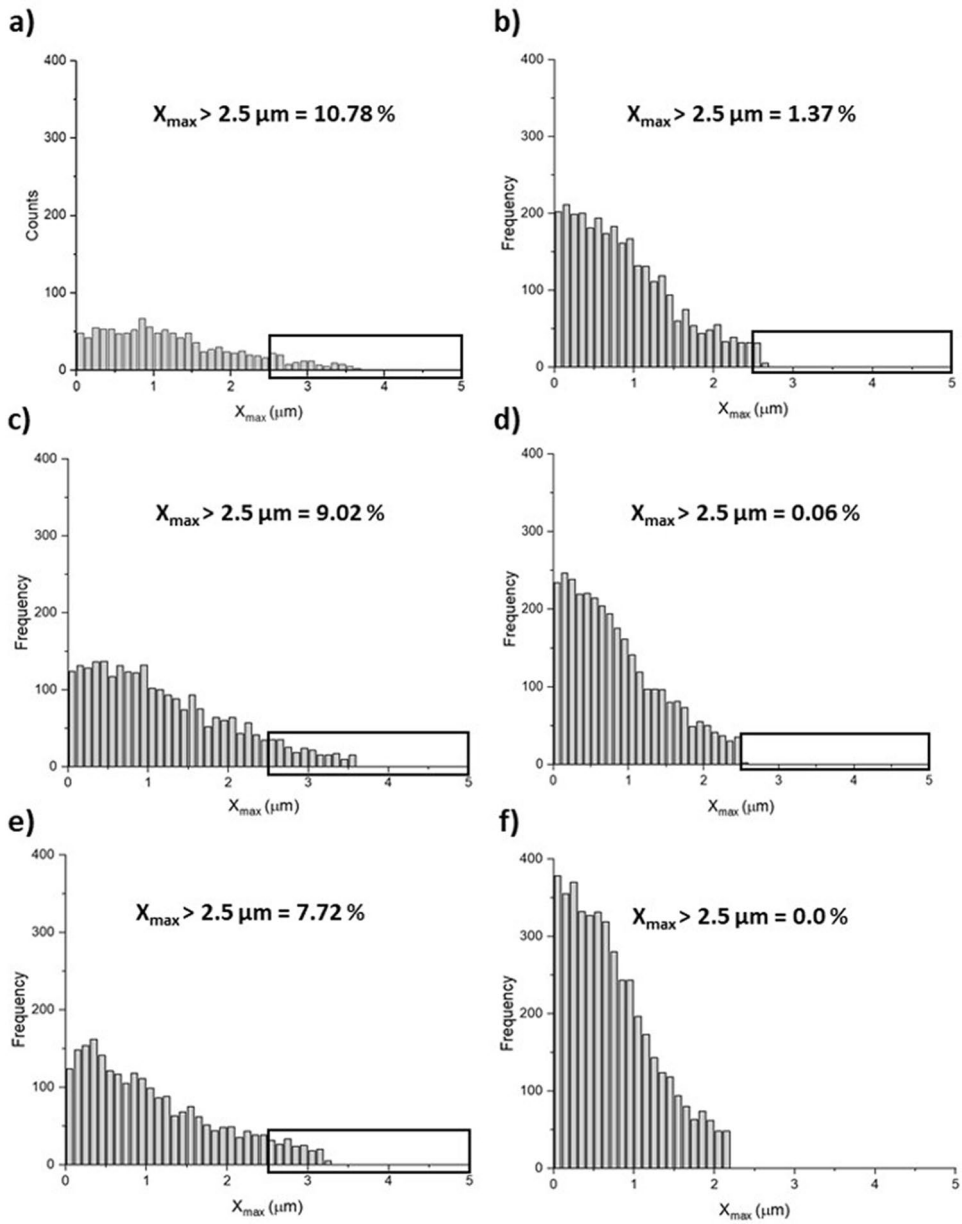
Showing histograms and percentages of Xmax > 2.5 µm for (**a**) HB-PNIPAM-Van-*S. aureus* at stage 1, (**b**) *S. aureus* at stage 1, (**c**) HB-PNIPAM-Van-*S. aureus* at stage 2, (**d**) *S. aureus* at stage 2, (**e**) HB-PNIPAM-Van-*S. aureus* at stage 3 and (**f**) *S. aureus* at stage 3.

HB-PNIPAM-Van-*S. aureus* aggregates exhibited far greater percentage of major unbinding events in the long range (% X_max_ > 2.5 µm) from the corneal surface compared to *S. aureus* aggregates only, at each temperature stage (*p* < 0.05) shonw in Table 1 with histograms shown in Fig. 5a–f. At stage 1 (22 °C, T_1_ < LCST), there are almost 10 × more events occurring beyond 2.5 µm z-length for HB-PNIPAM-Van-*S. aureus*, compared *S. aureus* aggregates only. This is then further increased to 100 × at stage 2 (40 °C, T_2_ > LCST) compared to *S. aureus* aggregates only. At stage 3 (22 °C, T_3_ < LCST), there are no unbinding events obtained after 2.5 µm z-length for *S. aureus* aggregates while HB-PNIPAM-Van-*S. aureus* aggregates show a slight reduction compared to those measured at stage 2 (40 °C, T_2_ > LCST). HB-PNIPAM-Van-*S. aureus* shows a reduction in X_max_ > 2.5 µm from 10.78% to 9.02% above the LCST from stage 1 to 2, with a subsequent 1.3% reduction at stage 3. *S. aureus* aggregates exhibited a 1.31% reduction in X_max_ > 2.5 µm from stage 1 to 2 above the LCST, with a subsequent 0.06% reduction below the LCST at stage 3.

## Discussion

A novel approach of AFM MBFS was developed to determine the quantitative mechanical properties and interactions of bacteria-polymer complexes on a corneal model, during unbinding, in-vitro. Through the use of temperature cells, the methodology was successfully applied during sequential temperature-dependent phase transitions above and below HB-PNIPAM’s LCST, enabling the effect of solvation phase transition on *S. aureus* aggregate detachment to be assessed.

It is evident from Table 1 that bacterial aggregates are present on the control and experimental group AFM probes compared to poly-DOPA only AFM probes at each temperature stage. This is due to *S. aureus* adhesive surface proteins^47^ being responsible for a 40-fold increase in energy dissipation upon removal from the corneal surface, compared to poly-DOPA only functionalised probes. HB-PNIPAM-Van has shown mechanical functionality in reducing the energy dissipation in *S. aureus* aggregate removal during the temperature transition regime. It is postulated that heating the environment surrounding the living probe to T > LCST in PBS shrinks the aggregate through polymer desolvation^40^, consequently reducing the size of the bacteria-polymer complex^22,35–37^. This may have led to a reduction in surface area contact^48^ between the functionalised probe and corneal surface, reducing the work done to remove it^41,48^. If no bound HB-PNIPAM-Van is present, the aggregate would not shrink, maintaining the surface area of contact and its energy dissipation upon removal, evident by no significant change in *S. aureus* aggregate energy dissipation at stage 1 to 2 (*p* > 0.05). From the energy dissipation data, it is evident that HB-PNIPAM-Van has a marked influence on the reduction of the removal energy of *S. aureus* aggregates through its thermo-active functionality^22,35–37^. Although, the significantly greater energy dissipation shown by HB-PNIPAM-Van-*S. aureus* compared to *S. aureus* aggregates at stage 1 and 2 may be a consequence of a larger aggregate being formed by the introduction of HB-PNIPAM-Van^22^. Thus, holding the bacteria-polymer complex together^22,35–37^. At Stage 3, a significant increase in energy dissipation was found for HB-PNIPAM-Van-*S. aureus* after reducing the temperature below the LCST*.* This may indicate HB-PNIPAMs functionality as reversible, although, this was also found for *S. aureus* aggregates (*p* < 0.05). The living probe may be experiencing probe death (attached bacterial death) after continuous use for over 2 h (30 min × 3, heating and cooling, and indentation protocol), as shown by Aguayo and co-workers using SCFS on Titanium implants^43^.

HB-PNIPAM-Van-*S. aureus* exhibited functionality again with a significant increase and subsequent decrease in breaking force during the temperature phase regime (*p* < 0.05). These breaking force values were found to be similar to non-specific adhesion values from the unbinding of *S. aureus* to Titanium implant surfaces^43^, nanopatterned polycarbonate surfaces^50^ and collagen^52^. As there was no significant change in breaking force during all temperature stages for *S. aureus* aggregates only shown in Table 1, the significant increase and subsequent decrease in breaking force can only be attributed the thermo-active phase transition of HB-PNIPAM-Van^22,35–37^.

Worm-like-chain analysis (WLC)^50,51,53^ was performed to quantify the unbinding events occurring during probe removal from the corneal surface. Previous work has shown utility in applying this model during single cell force spectroscopy (SCFS) of bacteria to clinical surfaces of interest^43,50,51,53^. The contour length given by the WLC model represents the total length of a completely, unfolded molecule or protein^58^. Here, we are assuming the contour length is the total length of the aggregate itself and there is no stretching of poly-DOPA or HBPNIPAM. While the WLC model is traditionally used in SCFS for the analysis of surface proteins from an individual bacterium, here it is a complex consisting of a large bacteria and small polymer portion. From this, we measure the interaction of the entire aggregate complex from the cornea. A z-length (probe removal length) of 5 µm is a very long-range interaction as typical SCFS interactions tend to be in the order of nanometers^43^. Corneal surfaces consist primarily of type 1 collagen^56^, which has a contour length between 14.5 and 309 nm^57^. The use of 5 µm unbinding length and contour length data shows that it is the aggregate complex (combination of bacteria and polymer) that is interacting with the cornea.

Changes in distances between unbinding events (ΔL) can be associated with HB-PNIPAM playing a significant role in holding the bacterial aggregate together during unbinding across the total probe retract z-length (5 µm). As shown in the energy dissipation results, no significant change in ΔL was found after transition back to T_3_ < LCST for HB-PNIPAM-Van-*S. aureus*. Again, indicating the living probe may be experiencing bacterial death after continuous use for over 2 hours^43^.

Contour lengths for HB-PNIPAM-Van-*S. aureus* aggregates were significantly larger than *S. aureus* only (*p* < 0.05), demonstrating the impact of HB-PNIPAM-Van maintaining the aggregate structure. HB-PNIPAM-Van showed functionality by significantly increasing and subsequently decreasing aggregate contour length during the temperature transition regime (*p* < 0.05). It was assumed that this would be the reverse case, with the HB-PNIPAM-Van-*S. aureus* contour length decreasing at T_2_ > LCST due to the deslovation associated phase transition, although this did not occur. It is postulated that the increase in breaking force during stage 2 for HB-PNIPAM-Van-*S. aureus* probes contributed to the increase in contour length. As the breaking force from the corneal surface was greater, this elongated the aggregate during removal, further evident by far greater percentage of unbinding events in the long range (% X_max_ > 2.5 µm) at T > LCST.

The number of fitted segments i.e. the total number of unbinding events in the retract curve was significantly less across all temperature stages for HB-PNIPAM-Van-*S. aureus* compared to *S. aureus* only (*p* < 0.05) shown in Table 1, with histograms shown in Fig. 4. This may indicate HB-PNIPAM-Van does not enable *S. aureus* bacteria to fully attach via its surface adhesins after 30 s of 5 nN indentation force, whereby, only the contact face portion of the aggregate (away from the probe) attaches to the cornea. During HB-PNIPAMs phase transition at stage 2 (T > LCST), the aggregate further contracts onto the probe surface, increasing probe coverage and hence increases the surface area of contact of probe to the corneal surface during indentation. It is postulate that this increase in contact area increases the number of unbinding events during probe removal as more surface adhesins attach. The percentage of fitted segments in the short range (% FSSR) was significantly lower for HB-PNIPAM-Van-*S. aureus* aggregates compared to *S. aureus* aggregates only, at stage 1 and 2 (*p* < 0.05), shown in Fig. 4a,c. This indicates that more surface adhesins were in contact during unbinding for *S. aureus* aggregates compared to HB-PNIPAM-Van-*S. aureus* aggregates. The percentage of fitted segments in the long range (% FSLR) were significantly greater for HB-PNIPAM-Van-*S. aureus* aggregates compared to *S. aureus* aggregates only, at stage 1 and 2 (*p* < 0.05), shown in Fig. 4a,c, indicative of tethering in the longer ranges by HB-PNIPAM-Van holding the aggregate together.

In this investigation, a clinical strain of *S. aureus* was used to model the effect a novel, non-AMR, polymer-based intervention (HB-PNIPAM-Van) has on bacterial aggregate unbinding to corneal surfaces*, *in-vitro. AFM SCFS and MBFS has provided utility in determining the unbinding characteristics of single bacteria^43,48,50–53^ and biofilms^44^. This approach, unlike SCFS and traditional MBFS, focuses more on bacterial aggregates as opposed to individual cells or whole biofilms. Due to the nature of this investigation, an intermediate approach between these two techniques was sought, whereby the numerous bacteria, either functionalised at the terminals of HB-PNIPAM-Van or not, could be attached and subsequently detached from a corneal apex. Using a relatively large spherical indenter functionalised with a bacterial binding agent poly-DOPA^43^, the unbinding assessment of bacterial aggregates to corneal surface was successfully conducted.

This study applied a living probe loading force of 5 nN to the corneal surfaces while previous SCFS studies applied much lower loading forces of 500 pN^43,50^. A 5 nN loading force was chosen to initiate a significant amount of bacterial aggregate contact to the corneal surface. When using multiple cells (10’s-100’s) on a larger spherical probe (50 µm), it was assumed compressive stress of the probe is much less than on a single cell. Particularly, when applied to a relatively soft substrate such as a cornea, as substrate deformation can also occur. Taking into consideration the mechanical compliance of the aggregates and cornea, increasing the contact force from 500 pN for effective unbinding measurements was explored and after several tests, 5 nN was used to ensure good contact (data not shown). Since the indentation force was normalised across all probes, the change in unbinding properties can be solely attributed to HBPNIPAM solvation.

Previous studies have also applied increasing dwell times up to 60 s to focus on the dynamics of adhesion as a function of contact time^43^, while others have used much lower dwell times of 0 s and 1 s^50^. This study focussed on a change of aggregate unbinding as a function of temperature, maintaining a dwell time of 5 s across all living probes contacting the corneal surface. While dwell times have shown to increase probe-surface adhesion^43,50^, from the data it was clear a dwell time of 5 s generated a significant amount of unbinding from the corneal surface, enabling the effect of thermo-active polymer solvation to be analysed. In the future, investigators may wish to incrementally increase or decrease the living probe dwell time for a clearer focus on the dynamics of aggregate adhesion.

When applying higher loads and intermediate surface dwell times on bacterial aggregate functionalised AFM probes, it is possible some bound bacteria may detach and remain on the corneal surface, after unbinding. Particularly, bacterial aggregates without the polymer binding components further holding the aggregate together, as demonstrated here. In future, investigators may wish to combine AFM with (top down) fluorescence imaging to detect aggregate remnants (individual bacteria) on the corneal surface, post detachment protocol. With previous studies showing *S. aureus* aggregate formation via fluorescence with the introduction of HB-PNIPAM-Van^22^, this would further highlight the effectiveness of HB-PNIPAM in bacterial aggregation and subsequent removal.

It is clear from the data that HB-PNIPAM exhibits functionality in aggregate removal during its temperature dependent phase transition^22^. It must be noted that infection of the corneal surface by *S. aureus* alone does not produce these aggregates and bacteria remain relatively dispersed^22^. It was assumed that non-specific adhesion data was not necessary to collect as no change in adhesive properties of individual bacteria were made, only the physical nature of the bacterial aggregate itself. Future investigations may wish to assess the effect of the polysaccharide capsule surrounding individual *S. aureus* bacteria^54^ via the use of genetic knockouts. Corneas extracted from healthy rabbits were placed in PBS solution and no surface modification was used such as the addition of ocular mucins^49^. It was assumed this would significantly impact the assessment of corneal-bacterial aggregate binding. In future, researchers should consider using ocular mucins for a more comprehensive in-vitro model, including the use of tear formulations^55^ instead of PBS.

With previous experiments assessing the aggregation properties of HB-PNIPAM-Van interventions on abraded corneal surfaces visually, this is the first study of its kind to assess the reversible mechanical impact on corneal detachment, in-vitro.

## Conclusion

This work has demonstrated the possibility of using AFM MBFS to determine the mechanical properties and unbinding characteristics of *S. aureus* aggregates to corneal surfaces and assessing the effect of phase transition temperature of HB-PNIPAM-Van-*S. aureus* during unbinding, in-vitro*,* through the use of temperature cells. The effect of temperature on the functionality of HB-PNIPAM-Van-*S. aureus* showed that the polymer-bacteria complex significantly reduced the work done in removing bacterial aggregates at T_2_ > LCST, exhibiting reversibility at T_3_ < LCST. While the breaking force increased at T_2_ > LCST, the number of unbinding events was significantly increased with the percentage of these occurring in the long range also increasing. The LCST phase transition temperature has also shown a 100 × more of these unbinding events occurring in the long-range z-length (> 2.5 µm) compared to *S. aureus* aggregates only. This study demonstrated a new technical approach to understand the effect of thermo-active polymers to reduce infection. This can now be used to examine the strength of binding of a range of other bacteria to both natural and synthetic substrates, information which will be fundamental to the design of new approaches to decrease surface infection without resorting to antibiotics.
